# Supplementary material for: Development and evaluation of a Physiotherapy-led, WHO-ICOPE-Based, Person-Centered Integrated Care Program (PTICOPE) module to enhance intrinsic capacity in older adults: Protocol for a randomized controlled trial
Source: PLoS One. 2025 Mar 19;20(3):e0318513. doi: 10.1371/journal.pone.0318513 (PMC11922222; doi:10.1371/journal.pone.0318513)
Supplement: S1 File — (PDF) [file pone.0318513.s001.pdf]

TCTR ID : TCTR20241029007

OTHER ID :

Overall Recruitment Status : Pending (Not yet recruiting)

Prospective registration
This protocol was registered before enrollment of the first participant.

Tracking Information

First Submitted Date : 27 October 2024

First Posted Date : 29 October 2024

Last Update Posted Date : 27 October 2024

Title

Public Title : Development of Physiotherapy-led Person-Centered Integrated Care For Older People based on the WHO-ICOPE and Assessment of its Impact on Intrinsic Capacity

Acronym : No Data

Scientific Title : Development and Evaluation of a Physiotherapy-led, WHO-ICOPE-Based, Person-Centered Integrated Care Program (PTICOPE) to Enhance Intrinsic Capacity in Older Adults: A Randomized Controlled Trial

Sponsor ID/ IRB ID/ EC ID : REC/09/2024 (PG/MR/437)

Registration Site : Thai Clinical Trials Registry

URL : https://www.thaiclinicaltrials.org/show/TCTR20241029007

Secondary ID : No Secondary ID

Ethics Review

1. Board Approval : Submitted, approved

Approval Number : REC/09/2024 (PG/MR/437)

Date of Approval : 03 September 2024

Board Name : UITM RESEARCH ETHICS COMMITTEE

Board Affiliation : Universiti Teknologi MARA Malaysia

Board Contact : Business Phone : 60355448069 Ext. No Data

Business Email : recsecretariat@uitm.edu.my

Business Address : Universiti Teknologi MARA, Aras 3, Bangunan Wawasan, 40450 Shah Alam, Selangor, Malaysia

Sponsor

Source(s) of Monetary or Material Supports : Faculty of Health Sciences, Universiti Teknologi MARA Selangor Branch

Study Primary Sponsor : Faculty of Health Sciences, Universiti Teknologi MARA Selangor Branch

Responsible Party : Name/Official Title : Maria Justine @ Stephany

Organization : Universiti Teknologi MARA

Phone : 60332584365 Ext. No Data

Email : maria205@uitm.edu.my

Study Secondary Sponsor : No Study Secondary Sponsor

Protocol Synopsis

Protocol Synopsis : The effects of Physiotherapy led Person Centered Integrated Care for Older People are understudied. This is a 12 weeks, multicenter, randomized controlled trial involving 70 community-dwelling older adults aged 60 to 75, recruited from Pusat Aktiviti Warga Emas (Activity Centers for Older People). Participants will be randomized to either the intervention group, receiving the PTICOPE workbook and guided use, or the control group, receiving general IC information. Data will be collected at baseline, 4th, 8th, and 12th weeks, to assess both primary and secondary outcomes. Primary outcomes include measures of locomotor, psychological, cognitive, vitality, visual, and hearing functions using validated scales. Secondary outcomes will evaluate quality of life, activities of daily living, urogenital health, and oral health. Data analysis will be conducted using SPSS version 29.

URL not available

Health Conditions

Health Condition(s) or Problem(s) Studied : Older adults who are living in the community, independent in activities of daily living may have declining intrinsic capacity (cognitive, locomotors, psychology, vitality, hearing, and vision).

Keywords : Community-dwellers, Intrinsic Capacity, Older adults

Eligibility

Inclusion Criteria : 1. Representative of the target population from Kedah state who visited the Pusat Aktiviti Wargae Emas (Centre for Senior CItizen).
2. Able to understand and communicate Bahasa Melayu and/ or English.
3. Older persons with the age range between 60-75 years old.
4. Individuals who is able to make decision on their own.
5. Individual who is willing to participate in the study.

Gender : Both

Age Limit : Minimum : 60 Years Maximum : 75 Years

Exclusion Criteria : 1. Limited Availability or commitment
2. Exclude individuals who hold biased or prejudiced views toward older adults or aging-related issues.
3. Communication and language issues.
4. Blind and deaf older person.

Accept Healthy Volunteers : Yes

Status

Overall Recruitment Status : Pending (Not yet recruiting)

Key Trial Dates		
	Study Start Date (First enrollment) : 01 November 2024	Indicate Type : Anticipated
	Completion Date (Last subject, Last visit) : 30 November 2026	Indicate Type : Anticipated
	Study Completion Date : 30 November 2026	Indicate Type : Anticipated

Design

Study Type : Interventional

Primary Purpose : Prevention

Study Phase : Phase 1

Intervention Model : Parallel

Number of Arms : 2

Masking : Open Label

Allocation : Randomized

Control : Active

Study Endpoint Classification : Efficacy Study

Sample size

Planned sample size : 70

Intervantion Arm 1

Intervention name : Physiotherapy-led Person-Centered Integrated Care for Older People (PTICOPE) based on the WHO-ICOPE framework

Intervention Type : Experimental

Intervention Classification : Behavioral

Intervention Description : The intervention for the PTICOPE study involves a 12 weeks, physiotherapy-led program aimed at enhancing intrinsic capacity in older adults, based on the WHO-ICOPE framework. Participants in the intervention group will receive a structured workbook with guided exercises and activities targeting key domains of intrinsic capacity, including locomotion, cognitive function, psychological well-being, vitality, vision, and hearing. Activities such as balance exercises, mindfulness practices, and cognitive tasks are included, alongside dietary and hydration guidance. The intervention is designed to be adaptable to individual needs, with personalized goal setting and progress tracking to support participants functional abilities and overall well-being.

Intervantion Arm 2

Intervention name : Control

Intervention Type : No Intervention

Intervention Classification : Behavioral

Intervention Description : The control group will receive a one-time health education session, covering general information on the importance of exercise, a healthy diet, and personal care to maintain intrinsic

capacity. Unlike the intervention group, the control group will not receive the PTICOPE workbook or personalized goal setting. This control setup is considered a no intervention comparator, as it does not include an active, ongoing intervention or tailored guidance like the PTICOPE program provided to the intervention group.

Outcome

Primary Outcome

1. Outcome Name : Intrinsic Capacity (Locomotor, Vitality, Psychological function, Cognitive Function, Hearing and Vision)
Metric / Method of measurement : Intrinsic capacity Screening tools by the World Health Organization
Time point : Baseline, week 4th, week 8th and week 12th

Secondary Outcome

1. Outcome Name : Instrumental Acitivities of daily Living
Metric / Method of measurement : Lawton-Brody Instrumental Activities of Daily Living Scale
Time point : Baseline and 12th week completion of intervention

2. Outcome Name : Quality of life
Metric / Method of measurement : Quality of Life Scale (QOLS)
Time point : Baseline and 12th week

3. Outcome Name : Urogenital Health
Metric / Method of measurement : Urogenital Distress Inventory Short Form (UDI-6)
Time point : Baseline and 12th week

Location

Section A : Central Contact

Central Contact
First Name : Nurhazrina | Middle Name : | Last Name : Noordin
Degree : Master in Physiotherapy | Phone : 60195285492 Ext. : No Data | Email : hazrinanoordin@yahoo.com

Central Contact Backup
First Name : Chye Wah | Middle Name : | Lastname : Yu
Degree : | Phone : 60175461957 Ext. : No Data | Email : chyewah@aimst.edu.my

Section B Facility Information and Contact

1. Site Name : Pusat Aktiviti Warga Emas (PAWE Sg Petani, PAWE Yan, PAWE Kulim)
City : Sg Petani | State/Province : Kedah | Postal Code : 08000
Country : Malaysia | Recruitment Status : Pending (Not yet recruiting)

Facility Contact
First Name : Chye Wah | Middle Name : | Last Name : Yu
Degree : PhD | Phone : 60175461957 Ext. : No Data | Email : chyewah@aimst.edu.my

Facility Contact Backup
First Name : Nurhazrina | Middle Name : | Last Name : Noordin
Degree : Master in Physiotherapy | Phone : 60195285492 Ext. : No Data | Email : hazrinanoordin@yahoo.com

Investigator Name
First Name : Maria | Middle Name : | Last Name : Justine
Degree : PhD | Role : Principal Investigator

Section C : Contact for Public Queries (Responsible Person)

First Name : Maria | Middle Name : | Last Name : Justine
Degree : PhD in GerontologyMaster in Physiotherapy | Phone : 60176573248 Ext. : No Data | Email : maria205@uitm.edu.my
Postal Address : Faculty of Health Sciences
State/Province : Selangor | Postal Code : 42300
Country : Thailand | Official Role : Study Principal Investigator
Organization Affiliation : Universiti Teknologi MARA

Section D : Contact for Scientific Queries (Responsible Person)

First Name : Maria | Middle Name : | Last Name : Justine
Degree : PhD in Gerontology | Phone : 60176573248 Ext. : No Data | Email : maria205@uitm.edu.my
Postal Address : Faculty of Health Sciences
State/Province : Selangor | Postal Code : 42300
Country : Thailand | Official Role : Study Principal Investigator
Organization Affiliation : Univerrsiti Teknologi MARA

Deidentified Individual Participant-level Data Sharing

Plan to share IPD : No

Reason : No plan of data sharing due to confidentiality of vulnerable subjects. However, sharing may be considered for certain request that is not against the principle of research ethics.

Publication from this study

MEDLINE Identifier : No Data

URL link to full text publication : No Data

This document was created on 18 November 2024 at 11:51 (GMT+7).
