# Supplementary material for: Development and evaluation of a Physiotherapy-led, WHO-ICOPE-Based, Person-Centered Integrated Care Program (PTICOPE) module to enhance intrinsic capacity in older adults: Protocol for a randomized controlled trial
Source: PLoS One. 2025 Mar 19;20(3):e0318513. doi: 10.1371/journal.pone.0318513 (PMC11922222; doi:10.1371/journal.pone.0318513)
Supplement: S2 File — (PDF) [file pone.0318513.s002.pdf]

STUDY PROTOCOL

Research Management Centre
Universiti Teknologi MARA
40450 SHAH ALAM

Application Form for Ethics Approval

Borang Permohonan Kelulusan Etika

This application is for the purpose of obtaining approval to conduct research.

Please attach a copy of Research Proposal.

Permohonan ini dikemukakan untuk tujuan kelulusan menjalankan penyelidikan.

Sila lampirkan salinan kertas cadangan penyelidikan.

Part A : Details of Researcher

Bahagian A: Maklumat Penyelidik

Title of Research Project :<br>*Tajuk Penyelidikan :*	Development of Physiotherapy-led Person-centered Integrated Care for Older People based on the WHO-ICOPE Framework and Assessment of its Impact on Intrinsic Capacity
Name of Researcher* :<br>*Nama Penyelidik*:*	Nurhazrina Binti Noordin
Name of Supervisor :<br>*Nama Penyelia*:	Assoc. Prof. Dr. Maria Justine @ Stephany
Address of Department/ Hospital/ Institute :<br>*Alamat Jabatan/ Hospital/ Institut:*	Centre for Physiotherapy Studies, Faculty of Health Sciences, UiTM, Puncak Alam Campus
Contact No/ Email :<br>*No.Telefon/ Emel :*	
Name of Study coordinator**:<br>*Nama Koordinator Kajian**:*	N/A
Contact No/ Email**:<br>*No.Telefon/ Emel**:*	N/A

☐ * Undergraduate / *Sarjana Muda*

☒ * Postgraduate / *Pasca Siswazah*

☐ * *Staff/Lecturers* / *Staf/Pensyarah*

☐ * External / *Pihak Luar*

** For Clinical Studies Only / *Untuk Kajian Klinikal Sahaja*

Does the research require an external Research Ethics Committee approval? (e.g. MREC)<br>Adakah penyelidikan ini memerlukan kelulusan Jawatankuasa Etika Penyelidikan Luaran? (contoh MREC)	
☐ Yes / *Ya*<br>☒ No / *Tidak*	

Research funding: Yes/ No – Awaiting approval from FRGS .
Dana Penyelidikan: Ada/ Tiada

If obtained, please complete section C.

Part B : Research Details
Bahagian B: Maklumat Penyelidikan

**Part B1** *Bahagian B1*	
☐ Interviews *Temubual*	☐ Case study *Kajian kes*
☐ Focus groups *Kumpulan focus*	☐ Clinical trial study *Kajian klinikal*
☐ Questionnaires *Soal selidik*	☒ Intervention study *Kajian intervensi*
☐ Action research *Kajian tindakan*	☐ Personal records *Rekod peribadi*
☒ Observation *Pemerhatian*	☐ Secondary data analysis *Analisis data sekunder*
	☐ Others (provide details): *Lain-lain (nyatakan):*

Part B2
Bahagian B2

1. Background:
(A brief explanation of the problem to be studied and literature review to support. Please append if more space is required)

Ageing populations is a national issues and it is also affecting developed countries such as Asian country. According to United Nations 2022, there are 761 million of older people all across the world and it was expected to reach 1603 million by 2050 across the globe. Comparing to the regions across the world, South East Asia shows prominent number of ageing populations and it is expected to be double the numbers by 2050 (United Nation, 2022). The population in WHO South-East Asia Region is ageing rapidly. While the proportion of people aged 60 or above was 9.8% in 2017, it will be increased to 13.7% and 20.3% by 2030 and by 2050, respectively (WHO,2024). Focusing on Malaysian populations, the ageing population was 7.4% in 2023 and it is expected to be 15% by 2050 (DOSM,2023).

As ageing approaches, health related issue includes intrinsic capacity (IC) decline will arise. IC refers to six main domains which is locomotors, psychological, cognitive, vitality, hearing and visual. Declining in these factors will give impacts on healthcare systems, economic productivity, social support systems, quality of life, psychological well-being, and policy planning from governments and organizations side. Prevalence of intrinsic capacity study in China reported a decline by 49.9% (Ma et al., 2021) indicating almost half of the aged population may have issues with daily lives. Another study conducted in 12 sites among 8 countries showed about 70% of the ageing populations presented with intrinsic capacity decline (Prince et al., 2021). While, a study conducted among 1000 participants in India reported about 84.5% IC decline, which mainly affected the locomotors and the least with vitality (Rao et al., 2023). The high IC decline reported in these studies highlight the importance of person-centered strategies and integrated care for older people through a multidisciplinary approach to promote the overall well-being.

Intrinsic capacity reflects individual physical and metal capabilities. According to Zhou and Ma (2022), older adults can achieve higher quality of life in their later years, when they are within suitable environment and reach the peak of each health phase, thus reducing the burden of the society. Intrinsic capacity consist of domains which includes locomotors (physical motilities and functional abilities), cognitive (mental functioning, including memory, reasoning and problem solving), psychological (emotional well-being and mental health status), sensory (vision and hearing capacities) and lastly vitality (overall energy and physiological vigor).

In 2017, the World Health Organizations (WHO) released a framework which is named as the Integrated Care for Older People (ICOPE) or also abbreviated as WHO-ICOPE. WHO-ICOPE is a framework to guide strategies to tackle ageing health issues that comprises the interactions of IC and functional ability (FA). Generally, the framework provides guidelines on a brief screening and pathway for managing older people with IC decline. Screening involves all IC domains and assess in greater depth will be done for conditions associated with loss in IC.Secondly a person centered assessment will be done and thirdly focus on develop a personalize care plan based on the screening process. Fourth steps involve ways of monitoring of the personalized care plan to monitor its effectiveness and lastly aim towards engaging with communities and caregivers. The framework offers a promising approached to address the complex healthcare needs of older people (WHO, 2017). ICOPE emphasize the importance of addressing the various domains of IC to promote a healthy ageing community. The pathway also includes the interactions with the environments and society which will be beneficial for the nation.

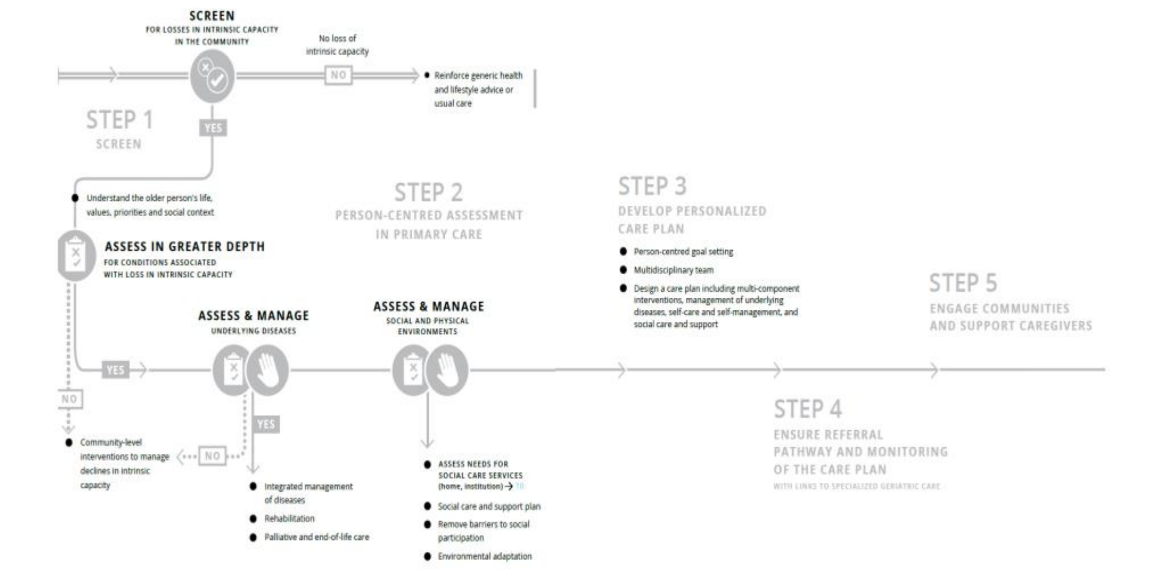

Figure 1.0 : WHO-ICOPE pathway

A few studies have used the ICOPE framework to the person-centered approach for older people. For instance, the AMICOPE (Alias et al., 2021), which have limitations in the lack of approach for the sensory domains. Another reported study known as the INSPIRE-ICOPE which targeted older people in the high income country with a decent level of education (Tavassoli et al., 2021), however, it may not be applicable in Malaysia due to diversity in the social and cultural background. Another study developed based on the ICOPE guideline also has some limitations as it targeted only on cognitive, locomotors and psychological domains (Casas-Herrero et al., 2022). Based on these limitations, further study is needed to incorporate all person-centered that may consist of engagement in

physical activity or exercises, advice and regular monitoring in dealing with all the IC domains. the domains

Problem statement:

Optimal intrinsic capacity (IC) is vital for older adults to age well, regardless of chronic illnesses or disabilities. Hence, healthcare providers need to design effective person-centered integrated care or interventions to improve functional abilities and overall well-being. Developing such interventions requires considering the diverse socioeconomic statuses, cultural backgrounds, and healthcare policies worldwide. Research has shown that factors influencing IC vary globally. For example, studies in China and Japan in 2021 indicated that IC decline is linked to social fragility and unmodifiable factors (Huang et al., 2021). In 2022, studies from India and China presented different findings on factors affecting IC decline. In India, age, gender, and smoking were found to negatively impact IC decline (Muneera et al., 2022), while in China, age and smoking were significant factors (Chen & Yang, 2022). Additionally, in India, IC decline was more closely related to chronic illness and healthcare quality. These findings suggest that factors contributing to IC decline vary by country due to sociocultural differences, necessitating tailored approaches to enhance IC in different regions.

The World Health Organization (WHO) released the ICOPE guideline, which outlines screening and management pathways for intrinsic capacity (IC) decline among older adults (WHO, 2017). This guideline also recommends a people-centered approach for effective screenings and interventions. Several studies have followed ICOPE recommendations, including AMICOPE, INSPIRE-ICOPE, and VIVIFRAIL. AMICOPE, an early tool with limitations, focuses on sensory domains (Alias et al., 2021); INSPIRE-ICOPE emphasizes technology and robotics (Tavassoli et al., 2021); and VIVIFRAIL covers all IC domains but mainly targets cognitive and locomotor functions. These studies demonstrate the varied approaches researchers have taken to develop IC interventions.

The differences in researchers' approaches to enhancing IC may be due to their interests, associated factors, and population demographics. Therefore, strategies to improve IC and functional abilities should be personalized based on individual goals. This ensures interventions are tailored to each person's needs. Additionally, older populations in Malaysia, particularly in rural areas, may have limited access to comprehensive healthcare services. Thus, a person-centered approach, adapting and adopting the ICOPE framework, could benefit tailored interventions led by physiotherapists. This study aims to develop a physiotherapy-led person-centered strategy to enhance all IC domains, promoting independence, functional abilities, and well-being among older adults. It also provides guidelines for physiotherapists working with older adults in the community to regularly screen IC and monitor outcomes.

References:
Rujukan:

1. Lee, S. B., Oh, J. H., Park, J. H., Choi, S. P., & Wee, J. H. (2018). Differences in youngest-old, middle-old, and oldest-old patients who visit the emergency department. Clinical and Experimental Emergency Medicine, 5(4), 249–255. https://doi.org/10.15441/ceem.17.261

2. Alias, S. B., Cuevas-Lara, C., Martínez-Velilla, N., Zambom-Ferraresi, F., Soto, M. E., Tavassoli, N., Mathieu, C., Heras Muxella, E., Garibaldi, P., Anglada, M., Amblàs, J., Santaeugènia, S., Contel, J. C., Domingo, À., & Casanovas, A. S. (2021). A multi-domain group-based intervention to promote physical activity, healthy nutrition and psychological wellbeing in older people with losses in intrinsic capacity: Amicope development study. International Journal of Environmental Research and Public Health, 18(11). https://doi.org/10.3390/ijerph18115979

3. Angioni, D., Nicolay, C., Vandergheynst, F., Baré, R., Cesari, M., & De Breucker, S. (2021). Intrinsic Capacity Assessment by a Mobile Geriatric Team During the Covid-19 Pandemic. Frontiers in Medicine, 8(May), 1–6. https://doi.org/10.3389/fmed.2021.664681

4. Beard, J. R., Jotheeswaran, A. T., Cesari, M., & Araujo De Carvalho, I. (2019). The structure and predictive value of intrinsic capacity in a longitudinal study of ageing. BMJ Open, 9(11). https://doi.org/10.1136/bmjopen-2018-026119

5. Belloni, G., & Cesari, M. (2019). Frailty and Intrinsic Capacity: Two Distinct but Related Constructs. Frontiers in Medicine, 6(June), 1–5. https://doi.org/10.3389/fmed.2019.00133

6. Carmel, S. (2019). Health and Well-Being in Late Life: Gender Differences Worldwide. Frontiers in Medicine, 6(October), 3–6. https://doi.org/10.3389/fmed.2019.00218

7. Casas-herrero, A., Anton-rodrigo, I., Zambom-ferraresi, F., Asteasu, M. L. S. De, Martinez-velilla, N., Elexpuru-estomba, J., Marin-epelde, I., & Ramon-espinoza, F. (2019). 13063_2019_Article_3426. 1–12.

8. Chan, Y. M., Ganapathy, S. S., Tan, L. A., Alias, N., Nasaruddin, N. H., & Khaw, W. F. (2022). The burden of premature mortality among older adults: a population-based study in Malaysia. BMC Public Health, 22(1), 1–8. https://doi.org/10.1186/s12889-022-13608-9

9. Chen, F., & Yang, M. (2022). Effects of personal and health characteristics on the intrinsic capacity of older adults in the community : A cross-sectional study using the healthy aging framework. 1–10.

10. Cheng, X., Yang, Y., Schwebel, D. C., Liu, Z., Li, L., Cheng, P., Ning, P., & Hu, G. (2020). Population ageing and mortality during 1990-2017: A global decomposition analysis. PLoS Medicine, 17(6), 1–17. https://doi.org/10.1371/journal.pmed.1003138

11. Chew, S. T. H., Kayambu, G., Lew, C. C. H., Ng, T. P., Ong, F., Tan, J., Tan, N. C., & Tham, S. L. (2021). Singapore multidisciplinary consensus recommendations on muscle health in older adults: assessment and multimodal targeted intervention across the continuum of care. BMC Geriatrics, 21(1), 1–14. https://doi.org/10.1186/s12877-021-02240-8

12. Drenth-van Maanen, A. C., Wilting, I., & Jansen, P. A. F. (2020). Prescribing medicines to older people—How to consider the impact of ageing on human organ and body functions. British Journal of Clinical Pharmacology, 86(10), 1921–1930. https://doi.org/10.1111/bcp.14094

13. Huang, C. H., Okada, K., Matsushita, E., Uno, C., Satake, S., Martins, B. A., & Kuzuya, M. (2021). The association of social frailty with intrinsic capacity in community-dwelling older adults: a prospective cohort study. BMC Geriatrics, 21(1), 1–11. https://doi.org/10.1186/s12877-021-02466-6

14. Jiang, Y. S., Shi, H., Kang, Y. T., Shen, J., Li, J., Cui, J., Pang, J., Zhang, C., & Zhang, J. (2023). Impact of age-friendly living environment and intrinsic capacity on functional ability in older adults: a cross-sectional study. BMC Geriatrics, 23(1), 1–9. https://doi.org/10.1186/s12877-023-04089-5

15. Holvoet, E., Wyngaert, K. Vanden, Van Craenenbroeck, A. H., Van Biesen, W., & Eloot, S. (2020). The screening score of Mini Nutritional Assessment (MNA) is a useful routine screening tool for malnutrition risk in patients on maintenance dialysis. PLoS ONE, 15(3), 1–13. https://doi.org/10.1371/journal.pone.0229722

16. Ismail, Z., Ahmad, W. I. W., Hamjah, S. H., & Astina, I. K. (2021). The impact of population ageing: A review. Iranian Journal of Public Health, 50(12), 2451–2460. https://doi.org/10.18502/ijph.v50i12.7927

17. Jayawardhana, T., Anuththara, S., Nimnadi, T., Karadanaarachchi, R., Jayathilaka, R., & Galappaththi, K. (2023). Asian ageing: The relationship between the elderly population and economic growth in the Asian context. PLoS ONE, 18(4 APRIL), 1–19. https://doi.org/10.1371/journal.pone.0284895

18. Jiang, X., Chen, F., Yang, X., Yang, M., Zhang, X., Ma, X., & Yan, P. (2023). Effects of personal and health characteristics on the intrinsic capacity of older adults in the community: a cross-sectional study using the healthy aging framework. BMC Geriatrics, 23(1), 1–10. https://doi.org/10.1186/s12877-023-04362-7

19. Leung, A. Y. M., Su, J. J., Lee, E. S. H., Fung, J. T. S., & Molassiotis, A. (2022). Intrinsic capacity of older people in the community using WHO Integrated Care for Older People (ICOPE) framework: a cross-sectional study. BMC Geriatrics, 22(1), 1–12. https://doi.org/10.1186/s12877-022-02980-1

20. Levy, T., Laver, K., Killington, M., Lannin, N., & Crotty, M. (2019). A systematic review of measures of adherence to physical exercise recommendations in people with stroke. Clinical Rehabilitation, 33(3), 535–545. https://doi.org/10.1177/0269215518811903

21. Livingston, G., Huntley, J., Sommerlad, A., Ames, D., Ballard, C., Banerjee, S., Brayne, C., & Burns, A. (2020). Since January 2020 Elsevier has created a COVID-19 resource centre with free information in English and Mandarin on the novel coronavirus COVID- research that is available on the COVID-19 resource centre - including this for unrestricted research re-use a. January.

22. Logan, A. C., Berman, B. M., & Prescott, S. L. (2023). Vitality Revisited : The Evolving Concept of Flourishing and Its Relevance to Personal and Public Health.

23. Löhler, J., Cebulla, M., Shehata-Dieler, W., Volkenstein, S., Völter, C., & Erik Walther, L. (2019). Schwerhörigkeit im Alter – Erkennung, Behandlung und assoziierte Risiken. Deutsches Arzteblatt International, 116(17), 301–310. https://doi.org/10.3238/arztebl.2019.0301

24. Lu, F., Li, J., Liu, X., Liu, S., Sun, X., & Wang, X. (2023). Diagnostic performance analysis of the Integrated Care for Older People (ICOPE) screening tool for identifying decline in intrinsic capacity. BMC Geriatrics, 23(1), 1–8. https://doi.org/10.1186/s12877-023-04180-x

25. Luis, J., Sánchez, S., Udina, C., Rincón, A. M., Victor, M. E., Martín, I. B., Cuesta, D. M., Epelde, I. M., Espinoza, F. R., Latorre, M. S., Idoate, F., Sarriés, A. G., & Martínez, B. M. (2022). Effect of a multicomponent exercise program and cognitive stimulation (VIVIFRAIL - COGN) on falls in frail community older persons with high risk of falls : study protocol for a randomized multicenter control trial. 1–15. https://doi.org/10.1186/s12877-022-03214-0

26. Mafauzy, M. (2000). The problems and challenges of the aging population of malaysia. The Malaysian Journal of Medical Sciences : MJMS, 7(1), 1–3. http://www.ncbi.nlm.nih.gov/pubmed/22844207%0Ahttp://www.pubmedcentral.nih.gov/articlerender.fcgi?artid=PMC3406209

2.

Research objectives:
Objektif penyelidikan:

1. To develop the physiotherapy-led person-centered Integrated Care for Older People (PTICOPE) (exercise, advice and monitoring interventions) based on a systematic review and WHO-ICOPE framework.

2. To validate the newly developed Physiotherapy-led Person-Centered Integrated Care for Older People (PTICOPE).

3. To determine the effects of PTICOPE on intrinsic capacity (cognitive, locomotor, psychology, vitality, hearing and vision) among older persons living in the community.

**3.**	Expected benefits:<br><br>1. The first objective it to focus on identifying the approaches done in previous studies which includes advice, interventions and monitoring from physiotherapy perspective on the positive impact on intrinsic capacity. It acts as a baseline to provide information and guideline before proceeding to development stage for the person-centered integrated care based on WHO-ICOPE framework. This will give a guideline and opportunities to combine targeted interventions which work with a holistic approach with multidisciplinary team in managing the intrinsic capacity decline among older people.<br><br>2. Then the process will continue with validation by healthcare professionals (content validation) and targeted participants (face validation). Content validation will provide feedback by the professionals in order for researcher to improve the approach before applying to the participants. Face validation will provide information on the suitability and understanding of the newly develop approach in terms of visual, instruction and exercise in the participant perspective.<br><br>3. The third objective is to determine the effects of the newly developed strategy (PTICOPE). Positive impacts of these approaches on managing the decline of intrinsic capacity will be beneficial and it acts as a guideline for healthcare professional in terms on personalized and targeted intervention for the older persons.
**4.**	Date of research commencement-end: Oct 2023 – November 2026
**5.**	Expected date of initial data collection: **July 2024**
**6.**	Location of research:<br><br>1. Pusat Aktiviti Warga Emas (PAWE) Sg. Petani<br>Daerah PKMD Kuala Muda<br><br>Kompleks Penyayang<br>Daerah Kuala Muda<br>08000 Kuala Muda, Kedah.<br><br>2. Pusat Aktiviti Warga Emas (PAWE) Yan<br>Daerah PKMD Yan<br>Kampung Belida 08800, Kedah.<br><br>3. Pusat Aktiviti Warga Emas (PAWE) Kulim<br>Daerah PKMD Kulim<br>Taman Mutiara Sungai Kob<br>09700 Kulim, Kedah.

**7.**	Research design dan methodology:<br>Rekabentuk penyelidikan dan metodologi:<br><br>**<u>STUDY 1: Research Objective 1</u>**<br><br>Study 1 aimed to develop a Physiotherapy-led Person-Centered Integrated Care for Older People (PTICOPE) via physiotherapy strategies or approaches based on the WHO-ICOPE framework and a systematic review.<br><br>A systematic review analysis aims to compile related studies on interventions to enhance IC and synthesize the information for the development of the PTICOPE.<br>Ethical Approval: Not applicable<br>Registration: PROSPERO.<br><br>Based on the WHO-ICOPE framework and findings from the systematic review, a PTICOPE strategies will be developed. The strategies will be in the form of a work-book that contains features such as:<br><br>1. Introductions<br>2. Baseline data collections<br>3. Intrinsic capacity screening.<br>4. Details of work-book<br>a) Objective<br>b) Referral person<br>c) Interventions<br>d) Monitoring diary<br>e) Advice for certain intrinsic capacity and home assessment.<br><br>The sample outlines/features of the proposed PTICOPE work-book will then undergo the validation process that consists of 2 phases which include content validation and face validation.<br><br>**<u>STUDY 2: Research Objective 2</u>**<br><br>**Phase 1(content validation) (Subject Info 1)**<br>1. Content validations will involve 12 healthcare professionals; occupational therapist, 2 physiotherapist practitioners, 2 physiotherapy lecturers, geriatrician, family medicine doctor, public health doctor, nutritionist, audiologists, psychologist and an international university collaborator.<br>2. An invitation (Information sheet and Consent form will be sent to the particular individuals, and if they agree to participate in these validation process, the researcher will send the work-book details together with PEMAT form to enable them in providing feedback towards the newly PTICOPE work-book.<br>3. The researcher expects to receive the feedback within 2 weeks and will do the necessary changes according to the feedback/comments.<br>4. Once the comments been received, amendments done accordingly and if necessary the amended copy will be sent back to the content experts for further comments. The process will continue until there are no further changes to be made.<br>5. The completed version will then undergo face validation.

Phase 2 (face validation) (Subject Info 2)

1. Face validation will involve 10 targeted participants from one of the PAWE listed in section 6.
2. An invitation (include information sheet and consent form) will be sent to the participants.
3. The researcher will gather the 10 participants in one of the PAWE (see section 6) in a meeting room. The researcher will present the contents of the PTICOPE work-book and will invite comments from the participants to gauge their understanding and acceptability of the contents of the PTICOPE work-book for visuals, understanding the task and the possibilities of the interventions to be done.
4. Two research assistants will be employed to help in the documentation of the discussion. At the same time, the discussion will be audio-recorded throughout, which later will only be used by the researcher to note down important points obtained from the participants and to double-check with the points documented by the research assistants.
5. Comments received will be utilized in making changes in the work-book so that it will be ready for feasibility testing among the older persons.

STUDY 3 : Research Objective 3

Study 3 aimed to determine the effects the newly developed PTICOPE on intrinsic capacity among the older persons living in the community.

Study design

This is a 6-week pre- and post-test interventional study. Participants will be assigned to either the intervention or control group via computer generated method. The intervention group will receive the PTICOPE work-book with comprehensive guides on how to use the work-book. The control group will be given a general knowledge about the importance of good IC and advice on how to enhance each domain of IC that may include exercises and advice to seek relevant healthcare services based on findings from baseline IC measurements.

Independent variable (IV)

The IV for this study is the person centered integrated care interventions, combining exercise interventions, advice and monitoring. It includes all intrinsic capacity domains and functional ability which is a physiotherapy-led that involves multidisciplinary team.

Dependent variable

1. Level of intrinsic capacity (psychological (GDS-15), vitality (MNA), cognitive (MMSE), hearing (able to hear tuning forks 512Hz), vision (level of Snellen chart), and locomotors (SPPB).
2. Measurement will be taken at baseline (prior to intervention) and at week 6th (post measurement).

Study setting

This study will be conducted at 3 PAWE as mentioned in section 6. These PAWE were selected for ease of recruitment as the participants who attended this centers are from the community. These centers represent the top 3 highest with 60-75 years old populations compared to other districts in Kedah (DOSM, 2023).

Study participants

A purposive sampling method will be used to recruit participants for this study based on the pre-determined inclusion and exclusion criteria.

Ethical consideration

1. Ethical approval involving human subjects will be obtained from the UiTM Research Ethics Committee. This study is considered the least risky as it does not involve any invasive procedures. Risk for technical is considered low as methodology may not involve advances technologies, but rather performance-based assessment. Assessing older people will be based on the functional performance that reflects their daily functioning. In terms of timing, it is considered as medium. This is due to researcher may need to recruit a large sample size with the potential of dropouts due to health related issues or even death. Apart from that, in terms of budget, it is low as this study may not involve expensive tools.
2. All volunteered participants will be briefly explained about the purpose, procedures, rights to withdraw and benefits of the study. Once participants agree to be the subjects of the study, they will be required to sign an informed consent form before data collection. A copy of the Subject Information sheet and a signed consent form (carbon copy) will be given to the participants. The original copy of the signed consent will be kept by the researcher for documentation purposes.

Study Variables and Instrumentations

Baseline data collections

1. Baseline demographic details include date of birth/age, gender, ethnic, marital status, living arrangement, living status, education level, income level and utilization of healthcare center.
2. Health details such as smoking status, history of surgery, history of fall, mobility status, walking aids, eye glasses, denture, pacemaker, hearing aids and comorbidities.
3. Anthropometry and body composition measures include body height (cm), Body weight (kg), body mass index (kg/ m^2), waist circumference (cm), waist to height ratio, muscle mass (kg) using bioelectrical impedance analysis, calf circumference (cm) and fat mass (%).

Intrinsic capacity

1. Locomotors will be assessed by Short Physical Performance Battery (SPPB). It consist of 3 sections which is balance, gait and chair stand test .Balance will be divided into bilateral stand, semi-tandem, and tandem standing. Participants needed to stand at least 10 second in order to gain points. Second is gait test which involves 4 meters walking and cut off point depend on time taken to finish the task. Lastly, chair stand test involves sit to stand activity that required them to finish the 5 repetitions. Sum of those 3 components will be tabulate and compared at the 6th weeks of interventions.
2. Psychological will be assessed by using short geriatric depression scale (GDS-15) as it is high in specificity and sensitivity. It contains of 15 questions and the cut off points are 0-4(normal), 5-8(mild depression), 9-11(moderate depression) and 12-15 (severe depression. Results will be compared between 1st week 6th weeks of interventions.
3. Vitality will be assessed by Mini Nutritional Assessments (MNA) . It consists of 7 items which involves of measurement of the circumference. The cut off points are 12-14(normal nutritional status), 8-11(at risk of malnutrition) and 0-7 (malnourish). Results will be compared between 1st week 6th weeks of interventions.
4. Cognitive will be assessed by using Mini Mental State Examinations (MMSE) which consists of items such as orientations, registration, attention and calculations, recall and language. Total score will be calculated and the cutoff points are 0-10(severe), 10-20(moderate), 20-25(mild) and 25-30 (questionably significant). Results will be compared between 1st week 6th weeks of interventions.
5. Visuals will be assessed by using Snellen charts**.** The charts contain 11 line and participants are required to stand in 6 meters distance and reading from the bottom line up to the visible line. The visible line will be note down and compared from the first week and final weeks (6th week).
6. Hearing will be assessed by using tuning forks 512 Hz and participants will be asked whether they able to hear it or not.

Functional ability (secondary outcomes)

1. Activity daily living will be assessed by Lawton – Brody Instrumental Activities of Daily Living Scale (I.A.D.L.) using for the first week itself.
2. Quality of life will be assessed using Quality of Life Scale (QOLS).

Participants recruitments

The recruitment process for participants in this study will be conducted through a multi-faceted approach across older persons living at those particular districts whom used to visit Pusat Aktiviti Warga Emas (PAWE) to ensure a diverse and representative sample of older persons with possibilities of declining in intrinsic capacity level. PAWE is a government organization under the management of Malaysian Department of Social Welfare which organizes the social activity involving B40 older persons group. Potential participants will be given consent form before screening and upon expressing interest participants, potential older person will undergo a preliminary screening to assess their eligibility based on inclusion and exclusion criteria. Inclusion criteria will be older person age from 60 -75 years old reside in community in Kuala Muda, Kulim and Yan under Kedah state, individual who is able to walk independently or partially dependent on assistive device, individual who undergo surgery more than 3 months' time,

comorbidities (hypertension, high cholesterol, arthritis, ischemic heart disease, diabetes type 1 and type 2, kidney disease, heart failure, depression, chronic obstructive pulmonary disease.), individual who able to understand ,read and converse either in Bahasa Malaysia or English, and Individual who is willing to participate in the study. Following the initial screening, eligible participants will be provided with detailed information about the study, including its objectives, procedures, and potential risks and benefits. Informed consent will be obtained from individuals willing to participate.
The process is as below:

Selection of participants

Purposive sampling method will be used based on our inclusion and exclusion criteria and consent form will be given for participant selection. Selected participants will be given second consent form before baseline data collection. They will be assigned into control and intervention group by simple randomization (lottery method)/block randomization (computer generated). The interventions group will receive the PTICOPE intervention with a work-book, while the control group will receive one-off information about the importance of IC and advice on exercise and seeking healthcare service based on the findings gathered during the baseline. Baseline data collection will be collected after second consent form which involves intrinsic capacity evaluation (locomotors, psychological, vitality, cognitive, hearing and visual), demographic details, clinical details, anthropometry and body composition details, functional fitness, intrinsic capacity fitness, and functional ability. Measurement of IC will be done at baseline and week 6th. Ice breaking will be done in order to reduce the gap relationship between researcher and participants and practicing to wear mask will be enforced during the sessions to prevent any possibilities of communicable disease. Briefing of interventions will be done by researcher towards participants and their carer in order for them to understand the purpose, how to perform exercise, recording the exercise done, as well as following the advice provided. Participants in the intervention group will be provided with the PTICOPE work-book that contains a monitoring diary, exercise interventions, goal-setting and advice on seeking other healthcare services. Q&A sessions will be done in order to clarify any doubt among participants and carer towards the interventions. Monitoring of the participants in the intervention group will be done once a week, either by face to face or phone calls.

Inclusion and exclusion criteria:
Kriteria kemasukan dan pengecualian:

Content Expert Validation

Inclusion criteria:
Kriteria kemasukan:

1. Subject matter expertise in each IC domains.
2. Professional experience in related field at least 5 years .
3. Experts who are affiliated with reputable institutions recognized by local and international goverment.
4. Experts who is available and willing to participate in the study.

Exclusion criteria:
Kriteria pengecualian:

1. Lack of relavant expertise.
2. Individuals with conflict of interest with the study.
3. Individual who is directly involve in designing the study.

Face Validation

Inclusion criteria:
Kriteria kemasukan**:**

1. Representative of the target population from Kedah state who visited PAWE.
2. Able to understand and communicate Bahasa Melayu and English.
3. Older persons with the age range between 60-75 years old.
4. Individuals who is able to make decision on their own.
5. Individual who is willing to participate in the study.

Exclusion criteria:
Kriteria pengecualian:

1. Limited availability or commitment
2. Exclude individuals who hold biased or prejudiced views toward older adults or aging-related issues.
3. Communication and language issues.
4. Blind and deaf older person.

Feasibility study

Inclusion criteria:
Kriteria kemasukan:

1. Older person age from 60 -75 years old.
2. Individual who is able to walk independently or partially dependent on assistive device.
3. Individual who undergo surgery more than 3 months' time.
4. Comorbidities (hypertension, high cholesterol, arthritis, ischemic heart disease, diabetes type 1 and type 2, kidney disease, heart failure, depression, chronic obstructive pulmonary disease.)
5. Individual who able to understand and converse either in Bahasa Malaysia or English.
6. Individual who is willing to participate in the study.

Exclusion criteria:
Kriteria pengecualian:

1. Individual with critical illness.
2. Alzheimer disease and dementia.
3. Blind and deaf individuals.

References:

Cao M, Tang Y, Li S, Zou Y. Effects of High-Intensity Interval Training and Moderate-Intensity Continuous Training on Cardiometabolic Risk Factors in Overweight and Obesity Children and Adolescents: A Meta-Analysis of Randomized Controlled Trials. Int J Environ Res Public Health. 2021 Nov 12;18(22):11905. doi: 10.3390/ijerph182211905. PMID: 34831659; PMCID: PMC8623248.

Domaradzki, J., Cichy, I., Rokita, A., & Popowczak, M. (2020). Effects of tabata training during physical education classes on body composition, aerobic capacity, and anaerobic performance of under-, normal-and overweight adolescents. International Journal of Environmental Research and Public Health, 17(3), 876. https://www.mdpi.com/1660-4601/17/3/876/pdf

Imanudin, I., & Sultoni, K. (2017, March). Tabata training for increasing aerobic capacity. In IOP Conference Series: Materials Science and Engineering (Vol. 180, No. 1, p. 012205). IOP Publishing. https://iopscience.iop.org/article/10.1088/1757-899X/180/1/012205/pdf

Kusuma, I. D. M. A. W. (2019). The influence of the differences within the preliminary vo2max level on the Tabata training results. Jurnal SPORTIF: Jurnal Penelitian Pembelajaran, 5(2), 327-341. https://ojs.unpkediri.ac.id/index.php/pjk/article/download/13490/1364

Li J, Cheng W, Ma H. A Comparative Study of Health Efficacy Indicators in Subjects with T2DM Applying Power Cycling to 12 Weeks of Low-Volume High-Intensity Interval Training and Moderate-Intensity Continuous Training. J Diabetes Res. 2022 Jan 13;2022:9273830. doi: 10.1155/2022/9273830. PMID: 35071605; PMCID: PMC8776485.

Setiawan, E., Iwandana, D. T., Festiawan, R., & Bapista, C. (2020). Improving handball athletes' physical fitness components through Tabata training during the outbreak of COVID-19. Jurnal SPORTIF: Jurnal Penelitian Pembelajaran, 6(2), 375-389. https://ojs.unpkediri.ac.id/index.php/pjk/article/download/14347/1692

Tabata, I. (2019). Tabata training: one of the most energetically effective high-intensity intermittent training methods. The Journal of Physiological Sciences, 69(4), 559-572. https://link.springer.com/article/10.1007/s12576-019-00676-7

**9**	Sample size:<br>Saiz sampel:<br><br>**<u>Content validation.</u>**<br>Content validity was further defined by as the degree to which the test measures the concept and the test's applicability to the aspects measured **(Thorndike-Christ (2014**).The recommended number of experts to review a tool varies from 2 to 20 individuals **(Armstrong et al., 2005)** and this study will utilize 12 professionals.<br><br>**<u>Face Validation</u>**<br>A study of validity was done among 25 adolescent and it shows an effectiveness of the questionnaire (**Mousazadeh et al., 2017).**Moreover another face validation done for a content and structure shows that validation via 10 participants shows an effective results **(Melissa et al., 2018).**Another study done for face validation of questionnaire and validated by 10 participants from the targeted group and the study concludes that 10 is enough to show ultimate result for face validation **(Dalawi et al., 2023).** Based on the most recent research, the face validity range will be 10–15 individuals. This study chose to utilize 10 participants as a reference from the most recent research.<br><br>**<u>Impact study</u>**<br>**Sample size: 70 participants for 2 groups (35 for each group)**<br><br>The G-power 3 was used to calculate the sample size. Considering drop-outs due to reasons such as withdrawal, refuse to continue, death, etc, 30% were increased to ensure the adequacy and rationality of the experimental data. So the final sample size is determined as N=70. The figure below shows the parameters used for the calculation.<br>• Total size per group is (n=27) according to G-Power, 30% dropout is equal to (n=8). Total number of sample per group will be 35). Total sample size for intervention and control group will be 70 (35 x 2 = 70).

**10.**	Research flowchart:<br>Carta alir penyelidikan:

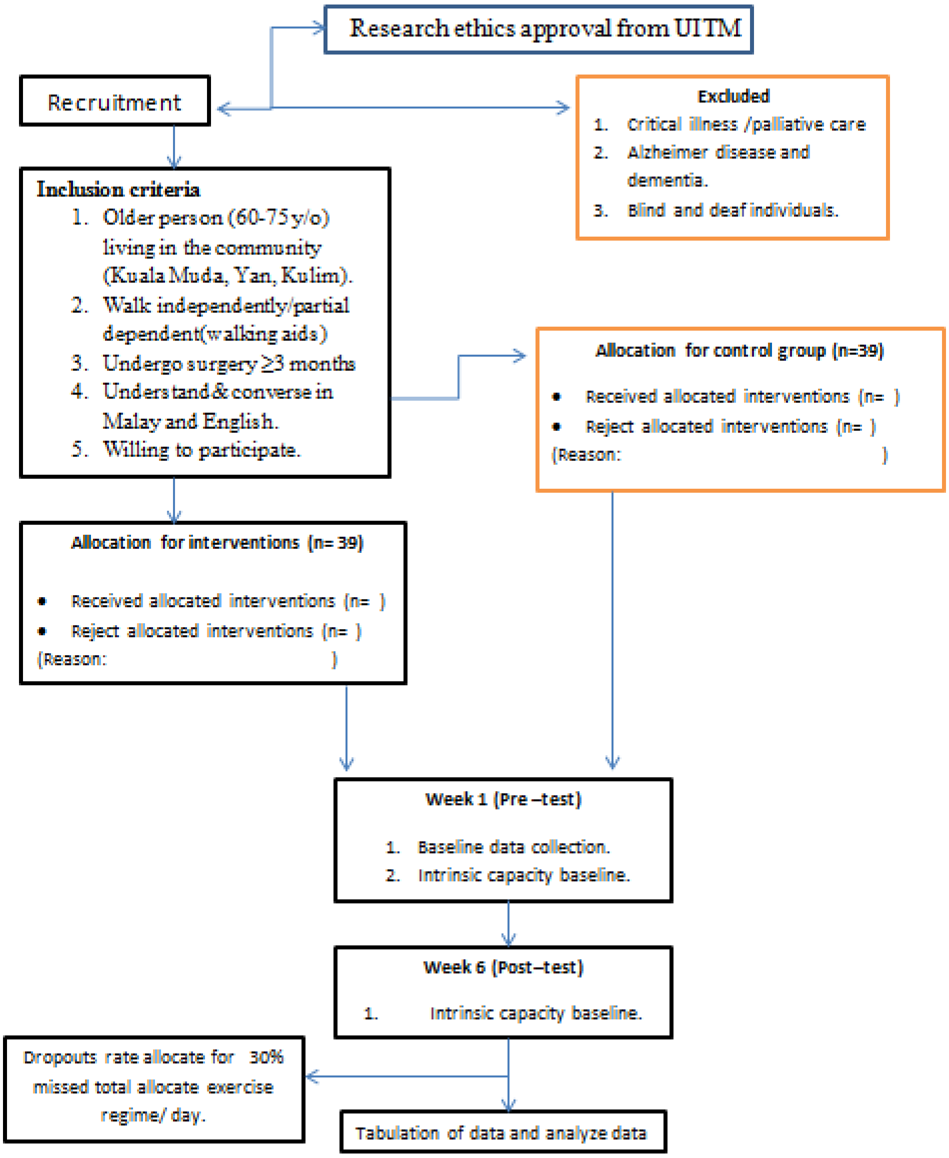

**11.**	Statistical analysis:<br><br>All statistical analyses will be conducted using SPSS version 29, where p<0.05 will be considered statistically significant. All data will be input into SPSS for further cleaning and analysis. To check for the presence of outliers and missing values, frequency distribution and graphical description will be established. To measure the distribution of the interval data, normality testing using the Kolmogorov-Smirnov test and Shapiro- Wilk tests will be used.<br><br>Descriptive analysis will be conducted using tables, averages, standard deviations, ranges, frequencies, and percentages to describe the background/characteristics of the study participants. Where necessary the statistical comparisons between the intervention and control groups will be performed using the independent t-test and Chi-square test.<br><br>To test the effects of the intervention on intrinsic capacity the paired t-test will be used to test the statistical significance in the changes of the IC.<br><br>Data storage: To maintain data privacy and confidentiality, after collecting hard-copy forms from participants, the datasets will be securely stored on password-protected laptops. Backup copies will be made on pen drives and hard drives, also password-protected. Only the principal researcher had access to these datasets.

*** If not applicable please write '-NA-' in the spaces provided.**
Jika tiada kaitan sila tulis '-NA-' di ruangan disediakan.

Part C: Funding details
Bahagian C: Maklumat Dana

**1.**	**Grant / Source:**<br>*Geran / Sumber:*	**Awaiting FRGS approval.**
**2.**	**Date of grant approval:**<br>*Tarikh kelulusan geran:*	**-NA-**
**3.**	**Total allocation:**<br>*Jumlah peruntukan:*	**-NA-**
**4.**	**Duration of grant:**<br>*Jangkamasa peruntukan:*	**-NA-**
**5.**	**Investigator services / professional fees:**<br>*Yuran perkhidmatan penyelidik / professional:*	**-NA-**
**6.**	**UiTM fees:**<br>*Yuran kepada UiTM:*	**-NA-**

**7.**	**Other facilities/resource provided by sponsoring organisation / company to investigator:**<br>*Lain-lain kemudahan / sumber disediakan organisasi penaja / syarikat kepada penyelidik:*	**-NA-**
**8.**	**Name and address of local sponsor / Clinical Research Organisation (CRO):**<br>*Nama dan alamat penyelidik tempatan / Organisasi Penyelidikan Klinikal (OPK) yang ditaja:*	**-NA-**

Part D: Agreement to conduct the research project.

Bahagian D: Pengesahan persetujuan menjalankan penyelidikan.

Must be completed and signed by all members of the research group.

Mesti dilengkapkan dan ditandatangani oleh semua ahli kumpulan penyelidikan.

1. Principal Researcher (to be filled by Academic Staf/Post-graduate Student only)
   Penyelidik utama (untuk dilengkapkan oleh Staf Akademik/Pelajar Pasca-siswazah sahaja)

**Name:**<br>*Nama:*	Nurhazrina Binti Noordin	
**Staff ID/Student ID:**<br>*No.Staf/No. Pelajar:*		
**Position/ Specialisation:**<br>*Jawatan/ Kepakaran:*	Post-graduate Student	
**Affiliation:**<br>*Jabatan:*	Centre for Physiotherapy Studies, Faculty of Health Sciences	
**Office:**<br>*Telefon pejabat:*	-NA-	
**Mobile phone:**<br>*Telefon bimbit:*		
**Email:**<br>*Emel:*		
**Signature:**<br>*Tandatangan:*		**Date:**<br>*Tarikh:*

2. Supervisor (If any)
   Penyelia (sekiranya ada)

**Name:**<br>*Nama:*	Assoc. Prof. Dr. Maria Justine @ Stephany	
**Staff ID/Student ID:**<br>*No.Staf/No. Pelajar:*		
**Position/ Specialisation:**<br>*Jawatan/ Kepakaran:*	Assoc Prof / Physiotherapy & Gerontology	
**Affiliation:**<br>*Jabatan:*	Centre for Physiotherapy Studies, Faculty of Health Sciences, UiTM, Puncak Alam Campus	
**Office:**<br>*Telefon pejabat:*		
**Mobile phone:**<br>*Telefon bimbit:*		
**Email:**<br>*Emel:*		
**Signature:**<br>*Tandatangan:*		**Date:**<br>*Tarikh:*

3. Co-Researcher

**Name:**<br>*Nama:*	Prof Dr. Yu Chye Wah	
**Staff ID/Student ID:**<br>*No.Staf/No. Pelajar:*		
**Position/ Specialisation:**<br>*Jawatan/ Kepakaran:*	**Position**<br>Professsor / Dean Faculty Of Allied Health Professions.<br>**Specialisation**<br>Rehabilitation sciences, Biostatistics,clinical measurement, emergency & critical care, health management, ergonomic risk assestment, education assestment & evaluation, pelvic-floor, ano-rectal disorder**.**	
**Affiliation:**<br>*Jabatan:*	Faculty Of Allied Health Professions, Asian Institute of Medicine, Science and Technology (AIMST).	
**Office:**<br>*Telefon pejabat:*		
**Mobile phone:**<br>*Telefon bimbit:*		
**Email:**<br>*Emel:*		
**Signature:**<br>*Tandatangan:*		**Date:**<br>*Tarikh:*

Part E: Verification from Faculty/State Research Committee
Bahagian E: Pengesahan Jawatankuasa Penyelidikan Fakulti/Negeri

We have deliberated on the application and propose as below:

Kami telah meneliti permohonan ini dan mencadangkan seperti di bawah:

[/] Minimal risk research. Recommend for approval without presentation.
Penyelidikan melibatkan risiko minima. Dicadangkan untuk mendapat kelulusan tanpa pembentangan.

[ ] More than minimal risk research. Recommend for approval with presentation.
Penyelidikan melibatkan risiko melebihi minima. Dicadangkan untuk mendapat kelulusan dengan pembentangan.

Comment if any:
Ulasan jika ada:

____

____

____

	PROF. MADYA DR. NAZRI CHE DOM<br>Pengerusi Ganti<br>Jawatankuasa Etika Penyelidikan<br>Fakulti Sains Kesihatan<br>UiTM Puncak Alam<br>Selangor	10/6/2024
**Signature** *Tandatangan:*<br>**Chair/Co-chair of Faculty/State Research Committee**<br>*Pengerusi/Pengerusi Ganti JK Penyelidikan Fakulti/Negeri*	**Official stamp:**<br>*Cop rasmi:*	**Date:**<br>*Tarikh:*

Research Risk Classification Form
Borang Klasifikasi Risiko Kajian

Title of Research Project: *Tajuk Penyelidikan:*	Development of Physiotherapy-led Person-centered Integrated Care for Older People based on the WHO-ICOPE Framework and Assessment of its Impact on Intrinsic Capacity.
Name of Researcher: *Nama Penyelidik:*	Nurhazrina Binti Noordin
Name of Supervisor: *Nama Penyelia:*	Assoc. Prof. Dr. Maria Justine @ Stephany
Address of Department and Hospital/ Institute : *Alamat Jabatan dan Hospital/ Institut:*	Centre for Physiotherapy Studies
Contact No/ E-mail : *No.Telefon/ Emel :*	

PLEASE ANSWER ALL QUESTIONS BELOW.
If your answer is '**Yes'** to any of the following questions, please include a brief information in the space provided.

SILA JAWAB KESEMUA SOALAN DI BAWAH.
*Sekiranya jawapan anda **'Ya'** kepada mana-mana soalan di bawah, sertakan maklumat ringkas di ruang yang disediakan.*

	**PARTICIPANT PROFILE**	No	Yes	Brief description
1.	Are the participants children (under 18 years old)? *Adakah peserta kanak-kanak (Umur di bawah 18 tahun)?*	√		
2.	Are the participants from a particular vulnerable group? (e.g. mental disorder, mentally challenged, disabled, minority, disadvantaged group etc.) *Adakah peserta daripada golongan rentan? (cth: kecelaruan mental, kelainan keupayaan intelektual, berkeperluan khas, minoriti dan sebagainya.)*	√		
3.	Are any of these participants/patients in terminal care? *Adakah peserta/pesakit ini memerlukan rawatan terminal?*	√		

4.	Are any of these participants unable or are incapable of giving consent? (i.e. consent will be obtained indirectly from a legal guardian etc.)<br>*Adakah peserta tidak boleh atau tidak berupaya memberi izin? (spt: izin akan diambil secara tidak langsung daripada penjaga sah dan sebagainya.)*	√		
5.	Are the participants given any form of emolument to participate?<br>*Adakah peserta diberi sebarang emolumen untuk menyertai kajian?*	√		

	**PRIVACY AND CONFIDENTIALITY**	No	Yes	Brief description
6.	Does any of the data collected have the potential to cause discomfort, embarrassment, or psychological harm to the participants? (e.g. sexual orientation etc.)<br>*Adakah data yang dikumpul berpotensi untuk menyebabkan ketidak selesaan, keaiban atau gangguan psikologi kepada peserta? (cth: orientasi seksual dan sebagainya.)*	√		
7.	Does your research involve measures undeclared to the participants? (e.g. covert observations etc.)<br>*Adakah penyelidikan anda melibatkan langkah-langkah yang tidak dimaklumkan kepada peserta? (cth: pemerhatian rahsia dan sebagainya.)*	√		
8.	Will the collected data be made available to other parties not involved in the research? (e.g. government agencies)<br>*Adakah data yang dikumpulkan akan didedahkan kepada pihak lain yang tidak terlibat dalam penyelidikan? (cth. agensi kerajaan)*	√		

	RISK OF HARM	No	Yes	Brief description
9.	Will you be collecting biological samples e.g. body fluids?<br>*Adakah anda akan mengumpul sampel biologi contohnya. cecair badan?*	√		
10.	Do you have access to any information that will allow the identification of individual human participants?<br>*Adakah anda mempunyai akses kepada apa-apa maklumat yang akan membolehkan pengenalpastian peserta secara individu?*	√		
11.	Is the collection method invasive and has the potential to cause harm, pain or discomfort?<br>(except finger, heel, ear prick.)<br>*Adakah kaedah pengumpulan invasif dan berpotensi menyebabkan kemudaratan, kesakitan atau ketidakselesaan?*<br>*(kecuali tusukan jari, tumit, telinga.)*	√		
12.	Will the participants be subjected to vigorous physical tests or exercise regime?<br>(if 'No', go to Question 15.)<br>*Adakah peserta akan melalui ujian fizikal atau senaman berintensiti tinggi?*<br>*(jika 'Tidak', teruskan ke Soalan 15.)*	√		
13.	Are the participants non-athletes or patients with chronic illness?<br>*Adakah peserta bukan atlet atau pesakit dengan penyakit kronik?*	√		
14.	Will they be subjected to maximal exercise intensity?<br>*Adakah mereka akan melalui senaman berintensiti maksimum?*	√		
15.	Is there any form of procedure/ medication involved?<br>*Adakah terdapat sebarang prosedur/ ubat yang terlibat?*	√		
16.	Is there any drug or device used with an unapproved indication?<br>*Adakah terdapat ubat atau peranti yang digunakan dengan tanpa indikasi yang diluluskan?*	√		
17.	Can the informed consent be obtained from anyone other than the patient/participants?<br>*Adakah keizinan kajian telah didapati daripada sesiapa selain pesakit/peserta?*	√		
18.	Is there any kind of risk to the participants if he/she chose to withdraw?	√		

	Adakah terdapat sebarang kemudaratan kepada peserta jika dia memilih untuk menarik diri?			
19.	Will the samples obtained be stored for future research?<br>*Adakah sampel yang dikumpul akan disimpan untuk penyelidikan di masa hadapan?*	√		
20.	Do you propose to analyse the sample outside of the original purpose for which it was collected?<br>*Adakah anda bercadang untuk menganalisa sampel selain tujuan asal ia dikumpulkan?*	√		
21.	If 'Yes' to No. 20, have you obtained consent from participants for this purpose?<br>*Jika 'Ya' pada No. 20, adakah anda mendapat persetujuan daripada peserta untuk tujuan ini?*			
22.	What type of biological samples collected?<br>(Please indicate amount and frequency.)<br>*Apakah jenis sampel biologi yang dikumpul?*<br>*(Sila nyatakan jumlah dan kekerapan.)*	√		

	**OTHER ETHICAL ISSUES**	No	Yes	Brief description
23.	Are there any other ethical issues not stated in this checklist?<br>*Adakah terdapat sebarang isu etika lain yang tidak dinyatakan dalam senarai semak ini?*	√		

Borang Maklumat Subjek

Tajuk Penyelidikan

PEMBANGUNAN STRATEGI BERPUSATKAN INDIVIDU YANG DITERAJUI OLEH FISIOTERAPI BERDASARKAN WHO-ICOPE DAN PENILAIAN KESANNYA TERHADAP KAPASITI INTRINSIK.

Pengenalan Penyelidikan

Penuaan penduduk adalah isu global yang memberi kesan besar kepada individu, masyarakat, dan sektor kerajaan. Salah satu kesan utama penuaan ialah penurunan kapasiti intrinsik, yang merangkumi enam domain utama: lokomotor, psikologi, kecergasan, kognitif, pendengaran, dan visual. Pada tahun 2017, Pertubuhan Kesihatan Sedunia (WHO) memperkenalkan rangka kerja yang menggabungkan kapasiti intrinsik dan keupayaan fungsi, dikenali sebagai Integrated Care for Older People (ICOPE). Namun, beberapa kajian terbaru yang menggunakan rangka kerja ICOPE mendapati ia tidak sesuai untuk digunakan di Malaysia.

Untuk mengatasi masalah ini, kajian ini sedang mengembangkan satu intervensi baru dalam bentuk buku kerja yang dipanggil Penjagaan Berpusatkan Individu Berintegrasi Untuk Orang Tua (PTICOPE). PTICOPE menggabungkan intervensi senaman, nasihat, dan pemantauan untuk warga emas yang dipimpin oleh ahli fisioterapi. Buku kerja ini merangkumi visual senaman dan carta pemantauan untuk membantu peserta memantau rutin harian mereka, termasuk senaman, pengambilan diet, dan pemantauan penilaian rumah. Selain itu, kajian ini juga bertujuan untuk menilai perubahan dalam tahap kapasiti intrinsik sebelum(pre-minggu pertama) dan selepas (post-minggu ke-enam) pelaksanaan PTICOPE pada peserta.

PTICOPE disasarkan kepada warga emas, melibatkan interaksi dan pemantauan berkala peserta bersama komuniti dan profesional penjagaan kesihatan. Ini dilakukan berdasarkan domain kapasiti intrinsik yang terjejas serta ketersediaan profesional penjagaan kesihatan yang berdekatan dengan kawasan kediaman peserta.

Tujuan Penyelidikan

Penjagaan Bersepadu Berpusat kepada Orang Tua (PTICOPE) adalah buku kerja yang dikembangkan oleh fisioterapi untuk mengatasi penurunan kapasiti intrinsik di kalangan warga emas. Sebelum PTICOPE diaplikasikan kepada peserta yang dipilih, kandungan buku kerja ini perlu disahkan oleh pakar. Matlamat utamanya adalah mendapatkan maklum balas mengenai buku kerja PTICOPE, yang merangkumi preskripsi latihan, nasihat, dan jadual pemantauan, daripada profesional penjagaan kesihatan pelbagai disiplin. Maklum balas dan komen yang diterima akan digunakan untuk memperbaiki PTICOPE sebelum ke tahap seterusnya.

Sebanyak dua belas profesional penjagaan kesihatan dari pelbagai disiplin dipilih untuk mengesahkan kandungan buku kerja ini. Mereka termasuk wakil dari WHO , terapi carakerja , ahli fisioterapi , pensyarah fisioterapi , pakar perubatan geriatrik , pakar perubatan keluarga , ahli dietetik , audiologis ,

psikologis/kaunselor , dan pensyarah universiti luar . Tujuan pengesahan ini adalah untuk memastikan kandungan buku kerja sesuai untuk digunakan oleh para peserta.

Prosedur Penyelidikan

Penyelidik akan menghantar surat jemputan berserta ringkasan tujuan penyertaan kepada anda. Sekiranya anda bersetuju untuk menyertai kajian ini sebagai pengesah kandungan penyelidik akan menghantar butiran buku kerja bersama-sama borang penilaian untuk membolehkan anda memberikan maklum balas terhadap buku kerja PTICOPE yang baru. Pengkaji menjangkakan akan menerima maklumbalas daripada anda dalam tempoh 2 minggu dan akan melakukan perubahan yang diperlukan mengikut maklum balas/komen. Setelah komen diterima, pindaan akan dilakukan sewajarnya jika perlu dan akan dihantar semula kepada pakar pengesah kandungan untuk ulasan lanjut. Proses akan diteruskan sehingga tiada lagi perubahan yang perlu dibuat. Versi yang telah siap kemudiannya akan menjalani pengesahan dari warga emas di komuniti.

Penyertaan dalam Penyelidikan

Penyertaan anda dalam kajian ini adalah secara sukarela. Anda boleh menolak untuk mengambil bahagian dan abaikan e-mel yang dihantar.

Faedah Penyelidikan

Maklumat yang diperoleh daripada pengesahan kandungan ini akan memberi manfaat terutamanya kepada profesional penjagaan kesihatan pelbagai disiplin dan warga emas kerana ia akan menyediakan garis panduan rejim senaman dan panduan preskripsi ini dapat dilakukan secara sistematik mengikut buku kerja PTICOPE.

Jika anda mempunyai sebarang soalan mengenai kajian ini atau hak anda, sila hubungi penyiasat di nombor telefon ()（Nurhazrina Binti Noordin) (Malaysia) ATAU Profesor Madya Dr Maria Justine () (Malaysia)

Risiko Penyelidikan

Tiada risiko yang diketahui berkaitan dengan penyelidikan ini terhadap semua pengesah kandungan. Masa adalah risiko utama bagi pengesah kandungan untuk menyelesaikan proses pengesahan.

Kerahsiaan

Maklumat anda akan dirahsiakan oleh penyiasat dan tidak akan didedahkan kepada umum melainkan pendedahan diperlukan oleh undang-undang.

Dengan menandatangani borang persetujuan ini, anda akan membenarkan semakan rekod, analisis dan penggunaan data yang timbul daripada penyelidikan ini.

Borang Persetujuan 1

Untuk menjadi subjek dalam penyelidikan, anda atau penjaga sah anda dikehendaki menandatangani Borang Persetujuan ini.

Saya dengan ini mengesahkan bahawa saya telah memenuhi syarat umur dan berkemampuan untuk bertindak bagi pihak saya sendiri / sebagai penjaga yang sah seperti berikut:

1. Saya memahami sifat dan skop penyelidikan yang dijalankan.
2. Saya telah membaca dan memahami semua terma dan syarat penyertaan saya dalam penyelidikan.
3. Semua soalan saya yang berkaitan dengan penyelidikan ini dan penyertaan saya di dalamnya telah dijawab dengan kepuasan saya.
4. Saya secara sukarela bersetuju untuk mengambil bahagian dalam penyelidikan ini, mengikuti prosedur kajian dan memberikan semua maklumat yang diperlukan kepada penyiasat seperti yang diminta.
5. Saya boleh pada bila-bila masa memilih untuk menarik diri daripada penyelidikan ini tanpa memberi sebarang sebab.
6. Saya telah menerima salinan Lembaran Maklumat Mata Pelajaran dan Borang Persetujuan.
7. Kecuali untuk kerosakan akibat daripada kecuaian atau kelakuan jahat penyelidik, saya dengan ini melepaskan dan melepaskan UiTM dan semua penyelidik yang mengambil bahagian daripada semua liabiliti yang berkaitan dengan, timbul daripada, atau berkaitan dengan penyertaan saya. Saya bersetuju untuk memastikan mereka tidak berbahaya daripada sebarang bahaya atau kerugian yang mungkin ditanggung oleh saya disebabkan penyertaan saya dalam penyelidikan.

Nama Subjek/Wakil yang diberi kuasa secara sah (LAR) Tandatangan

IC

Tarikh

Nama Saksi[3] Tandatangan

IC

Tarikh

Nama Pengambil Persetujuan Tandatangan

IC

Tarikh

1 Salinan asal yang ditandatangani hendaklah disimpan oleh Ketua Penyiasat.
2 Padamkan mana-mana yang tidak berkenaan.
3 Seorang saksi hanya diperlukan untuk persetujuan lisan

Subject Information Sheet

Research Title

Development of Physiotherapy-led Person-centered Strategy of integrated care for older people based on the WHO-ICOPE framework and Assessment of its Impact on Intrinsic Capacity

Introduction of Research

Aging populations are a global issue that significantly impacts individuals, communities, and government sectors. One of the main effects of aging is the decline in intrinsic capacity, which encompasses six major domains: locomotors, psychological, fitness, cognitive, auditory, and visual. In 2017, the World Health Organization (WHO) introduced a framework that integrates intrinsic capacity and functional ability, known as Integrated Care for Older People (ICOPE). However, several recent studies using the ICOPE framework have found it unsuitable for use in Malaysia.

To address this issue, this study is developing a new intervention in the form of a workbook called Integrated Person-Centered Care for Older People (PTICOPE). PTICOPE combines exercise interventions, advice, and monitoring for the elderly, led by a physiotherapist. This workbook includes exercise visuals and monitoring charts to help participants track their daily routines, including exercise, dietary intake, and home assessment monitoring. Additionally, this study aims to assess changes in the level of intrinsic capacity before (pre-first week) and after (post-sixth week) the implementation of PTICOPE on participants.

PTICOPE is targeted at the elderly, involving regular interaction and monitoring of participants with the community and healthcare professionals. This is done based on the affected domains of intrinsic capacity and the availability of healthcare professionals near the participants' residential areas.

Purpose of Research

Person-centered Integrated Care for Older People (PTICOPE) is a physiotherapy-led work-book targeted towards older person's intrinsic capacity level. Before implementing it towards older person participants, content expert validations should be obtained. Main target is to get feedback for our PTICOPE work-book which consist of exercise prescriptions, advice and monitoring charts from the multidisciplinary healthcare professionals as feedback and comments will be beneficial for amendments on our interventions before moving to the next phase. Twelve selected healthcare professional from multidisciplinary team includes WHO , occupational physiotherapist, physiotherapist practitioners , physiotherapy lecturers, geriatrician, family medicine , nutritionist , audiologist , psychologist/counselor , and university collaboration will be selected to be selected as a participants for content validation experts in this study.

Research Procedure

The researcher will send an invitation letter along with a summary of the purpose of participation to you. If you agree to participate in this study as a content validator, the researcher will send the work-book details along with the evaluation form to enable you to provide feedback on the new PTICOPE work-

book. The researcher expects to receive feedback from you within 2 weeks and will make the necessary changes according to the feedback/comments. Once the commends been received, amendments done accordingly if necessary will be sent back to the content experts for further comments. The process will continue until there are no further changes to be made. The completed version will then undergo face validation among the older people in the community.

Participation in Research

Your participation in this study is entirely voluntary. You may refuse to participate and overlook the mail.

Benefit of Research

The information obtained from this content validation will benefit mainly multidisciplinary healthcare professionals and older persons as it will provide guideline of the exercise regime and prescribing interventions for the older person will be systematically done according to the PTICOPE work-book.

If you have any question about this study or your rights, please contact the investigators at telephone number () （Nurhazrina Binti Noordin) OR Associate Professor Dr Maria Justine ().

Research Risk

There are no known risks associated with this research towards all of content validators. Time consuming is the main risk for the content validators to complete the validation process.

Confidentiality

Your information will be kept confidential by the investigators and will not be made public unless disclosure is required by law.

By signing this consent form, you will authorize the review of records, analysis and use of the data arising from this research.

Consent Form[1]

To become a subject in the research, you or your legal guardian is required to sign this Consent Form.
I herewith confirm that I have met the requirement of age and am capable of acting on behalf of myself / as[2] a legal guardian as follows:

1. I understand the nature and scope of the research being undertaken.
2. I have read and understood all the terms and conditions of my participation in the research.
3. All my questions relating to this research and my participation therein have been answered to my satisfaction.
4. I voluntarily agree to take part in this research, to follow the study procedures and to provide all necessary information to the investigators as requested.
5. I may at any time choose to withdraw from this research without giving any reason.
6. I have received a copy of the Subjects Information Sheet and Consent Form.
7. Except for damages resulting from negligent or malicious conduct of the researcher(s), I hereby release and discharge UiTM and all participating researchers from all liability associated with, arising out of, or related to my participation. I agree to hold them harmless from any harm or loss that may be incurred by me due to my participation in the research.

______________________________ Name of Subject/Legally authorized representative (LAR) Signature

______________________________ I.C No Date

______________________________ Name of Witness[3] Signature

______________________________ I.C No Date

______________________________ Name of Consent Taker Signature

______________________________ I.C No Date

[1] Original signed copy is to be retained by the Principal Investigator.
[2] Delete whichever is not applicable.
[3] A witness is only required for oral consent.

Borang Maklumat Subjek

Tajuk Penyelidikan

PEMBANGUNAN STRATEGI BERPUSATKAN INDIVIDU YANG DITERAJUI OLEH FISIOTERAPI BERDASARKAN WHO-ICOPE DAN PENILAIAN KESANNYA TERHADAP KAPASITI INTRINSIK.

Pengenalan Penyelidikan

Penuaan penduduk adalah isu global yang memberi kesan besar kepada individu, masyarakat, dan sektor kerajaan. Salah satu kesan utama penuaan ialah penurunan kapasiti intrinsik, yang merangkumi enam domain utama: lokomotor, psikologi, kecergasan, kognitif, pendengaran, dan visual. Pada tahun 2017, Pertubuhan Kesihatan Sedunia (WHO) memperkenalkan rangka kerja yang menggabungkan kapasiti intrinsik dan keupayaan fungsi, dikenali sebagai Integrated Care for Older People (ICOPE). Namun, beberapa kajian terbaru yang menggunakan rangka kerja ICOPE mendapati ia tidak sesuai untuk digunakan di Malaysia.

Untuk mengatasi masalah ini, kajian ini sedang mengembangkan satu intervensi baru dalam bentuk buku kerja yang dipanggil Penjagaan Berpusatkan Individu Berintegrasi Untuk Orang Tua (PTICOPE). PTICOPE menggabungkan intervensi senaman, nasihat, dan pemantauan untuk warga emas yang dipimpin oleh ahli fisioterapi. Buku kerja ini merangkumi visual senaman dan carta pemantauan untuk membantu peserta memantau rutin harian mereka, termasuk senaman, pengambilan diet, dan pemantauan penilaian rumah. Selain itu, kajian ini juga bertujuan untuk menilai perubahan dalam tahap kapasiti intrinsik sebelum(pre-minggu pertama) dan selepas (post-minggu ke-enam) pelaksanaan PTICOPE pada peserta.

PTICOPE disasarkan kepada warga emas, melibatkan interaksi dan pemantauan berkala peserta bersama komuniti dan profesional penjagaan kesihatan. Ini dilakukan berdasarkan domain kapasiti intrinsik yang terjejas serta ketersediaan profesional penjagaan kesihatan yang berdekatan dengan kawasan kediaman peserta.

Tujuan Penyelidikan

Tujuan penyelidikan ini adalah untuk mendapatkan pengesahan muka (face validation) bagi buku kerja PTICOPE daripada warga emas di komuniti. Matlamat utamanya adalah mengumpulkan maklum balas dan pandangan beberapa warga emas terpilih tentang buku kerja PTICOPE, yang mencakup preskripsi latihan, nasihat, dan jadual pemantauan, serta menilai kesesuaian dan pemahaman mereka tentang aktiviti senaman. Pengesahan ini melibatkan pengumpulan maklum balas daripada peserta yang mengunjungi Pusat Aktiviti Warga Emas (PAWE). Sasaran utama adalah mengintegrasikan cadangan dan maklum balas daripada peserta terhadap PTICOPE, menilai kesesuaian kandungan dan visual senaman, serta penerapannya dalam rutin harian mereka.

Prosedur Penyelidikan

Penyelidik akan menghantar surat jemputan berserta dengan ringkasan tujuan penyertaan dalam kajian ini. Sekiranya anda bersetuju sebagai peserta pengesah kandungan (face validator) anda akan diberikan borang persetujuan untuk ditandatangani dan seterusnya akan mendapat buku kerja PTICOPE. Penyelidik akan menjemput anda untuk berkumpul dalam bilik mesyuarat di salah satu pusat aktiviti warga emas yang terpilih. Penyelidik akan membentangkan kandungan buku kerja PTICOPE dan akan menjemput para peserta untuk memberi komen untuk mengukur pemahaman dan penerimaan mereka terhadap kandungan buku kerja PTICOPE mengenai visual, memahami tugas dan keberkesanan intervensi yang akan dilakukan. Dua orang pembantu penyelidik akan diambil bekerja untuk membantu dalam dokumentasi perbincangan. Pada masa yang sama, perbincangan akan dirakam secara audio sepanjang sesi perbincangan, yang kemudiannya hanya akan digunakan oleh penyelidik untuk mencatat perkara penting yang diperoleh daripada peserta dan menyemak semula dengan perkara yang didokumentasikan oleh pembantu penyelidik. Komen yang diterima akan digunakan dalam membuat perubahan dalam buku kerja supaya ia bersedia untuk ujian kebolehlaksanaan di kalangan warga emas yang terpilih untuk fasa seterusnya. Keseluruhan proses ini akan mengambil masa lebih kurang 1 ke 2 jam sahaja.

Penyertaan dalam Penyelidikan

Penyertaan anda dalam kajian ini adalah secara sukarela. Anda boleh menolak untuk mengambil bahagian dalam kajian atau anda boleh menarik diri daripada penyertaan dalam kajian pada bila-bila masa tanpa penalti.

Faedah Penyelidikan

Maklumat yang diperoleh daripada pengesahan ini akan memberi manfaat kepada peserta sebenar dari segi kebolehpraktisan buku kerja PTICOPE. Ia juga akan menyediakan garis panduan rejim senaman dan nasihat untuk menggalakkan komuniti penuaan yang sihat.

Jika anda mempunyai sebarang soalan mengenai kajian ini atau hak anda, sila hubungi penyiasat di nombor telefon ()（Nurhazrina Binti Noordin) ATAU Profesor Madya Dr Maria Justine ().

Risiko Penyelidikan

Tiada risiko yang diketahui berkaitan dengan penyelidikan ini terhadap semua pengesah kandungan.Masa adalah risiko utama bagi pengesah untuk menyelesaikan proses pengesahan.

Kerahsiaan

Maklumat anda akan dirahsiakan oleh penyiasat dan tidak akan didedahkan kepada umum melainkan pendedahan diperlukan oleh undang-undang.

Dengan menandatangani borang persetujuan ini, anda akan membenarkan semakan rekod, analisis dan penggunaan data yang timbul daripada penyelidikan ini.

Borang Persetujuan 1

Untuk menjadi subjek dalam penyelidikan, anda atau penjaga sah anda dikehendaki menandatangani Borang Persetujuan ini.

Saya dengan ini mengesahkan bahawa saya telah memenuhi syarat umur dan berkemampuan untuk bertindak bagi pihak saya sendiri / sebagai penjaga yang sah seperti berikut:

1. Saya memahami sifat dan skop penyelidikan yang dijalankan.
2. Saya telah membaca dan memahami semua terma dan syarat penyertaan saya dalam penyelidikan.
3. Semua soalan saya yang berkaitan dengan penyelidikan ini dan penyertaan saya di dalamnya telah dijawab dengan kepuasan saya.
4. Saya secara sukarela bersetuju untuk mengambil bahagian dalam penyelidikan ini, mengikuti prosedur kajian dan memberikan semua maklumat yang diperlukan kepada penyiasat seperti yang diminta.
5. Saya boleh pada bila-bila masa memilih untuk menarik diri daripada penyelidikan ini tanpa memberi sebarang sebab.
6. Saya telah menerima salinan Lembaran Maklumat Mata Pelajaran dan Borang Persetujuan.
7. Kecuali untuk kerosakan akibat daripada kecuaian atau kelakuan jahat penyelidik, saya dengan ini melepaskan dan melepaskan UiTM dan semua penyelidik yang mengambil bahagian daripada semua liabiliti yang berkaitan dengan, timbul daripada, atau berkaitan dengan penyertaan saya. Saya bersetuju untuk memastikan mereka tidak berbahaya daripada sebarang bahaya atau kerugian yang mungkin ditanggung oleh saya disebabkan penyertaan saya dalam penyelidikan.

____

Nama Subjek/Wakil yang diberi kuasa secara sah (LAR) Tandatangan

____ IC

Tarikh

____

Nama Saksi[3] Tandatangan

____ IC

Tarikh

____

Nama Pengambil Persetujuan Tandatangan

____ IC

Tarikh

1 Salinan asal yang ditandatangani hendaklah disimpan oleh Ketua Penyiasat.
2 Padamkan mana-mana yang tidak berkenaan.
3 Seorang saksi hanya diperlukan untuk persetujuan lisan.

Subject Information Sheet

Research Title

Development of Physiotherapy-led Person-centered Strategy of integrated care for older people based on the WHO-ICOPE framework and Assessment of its Impact on Intrinsic Capacity

Introduction of Research

Aging populations are a global issue that significantly impacts individuals, communities, and government sectors. One of the main effects of aging is the decline in intrinsic capacity, which encompasses six major domains: locomotors, psychological, fitness, cognitive, auditory, and visual. In 2017, the World Health Organization (WHO) introduced a framework that integrates intrinsic capacity and functional ability, known as Integrated Care for Older People (ICOPE).

However, several recent studies using the ICOPE framework have found it unsuitable for use in Malaysia. To address this issue, this study is developing a new intervention in the form of a workbook called Integrated Person-Centered Care for Older People (PTICOPE). PTICOPE combines exercise interventions, advice, and monitoring for the elderly, led by a physiotherapist. This workbook includes exercise visuals and monitoring charts to help participants track their daily routines, including exercise, dietary intake, and home assessment monitoring. Additionally, this study aims to assess changes in the level of intrinsic capacity before (pre-first week) and after (post-sixth week) the implementation of PTICOPE on participants.

PTICOPE is targeted at the elderly, involving regular interaction and monitoring of participants with the community and healthcare professionals. This is done based on the affected domains of intrinsic capacity and the availability of healthcare professionals near the participants' residential areas.

Purpose of Research

The purpose of this research is to obtain face validation for the PTICOPE workbook among older people in the community. The primary goal is to gather feedback and perspectives from selected older individuals on the PTICOPE work-book, which includes exercise prescriptions, advice, and a monitoring schedule regarding their suitability and understanding of the exercise activities and other contents. This validation is a process of obtaining feedback from targeted participants visiting the Elderly Activity Center (PAWE). The main objective is to integrate suggestions and feedback from participants on PTICOPE, whether it is suitable in terms of content and exercise visuals to be used as their daily routine.

Research Procedure

The researcher will send an invitation letter along with a summary of the purpose of participation in this study. If you agree as a content validator (face validator), you will be given a consent form to sign and then will receive the PTICOPE workbook. The researcher will invite you to gather in a meeting room at one of the selected elderly activity centers. The researcher will present the content of the PTICOPE workbook and will invite the participants to comment to measure their understanding and acceptance of the PTICOPE workbook content regarding visuals, task comprehension, and the effectiveness of the intervention to be carried out. Two researcher assistants will be employed to help in the documentation of the discussion. At the same time, the discussion will be audio-recorded throughout, which later will

only be used by the researcher to note down important points obtained from the participants and to double-check with the points documented by the research assistants. Comments received will be utilized in making changes in the work-book so that it will be ready for feasibility testing among the older persons. The whole procedure will take about 1 to 2 hours to complete.

Participation in Research

Your participation in this study is entirely voluntary. You may refuse to take part in the study or you may withdraw yourself from participation in the study at any time without penalty.

Benefit of Research

The information obtained from this face validation will benefit the participants in terms of the practicability of the PTICOPE work-book. It will also provide guideline of the exercise regime and advice in order to promote a healthy ageing community.

If you have any question about this study or your rights, please contact the investigators at telephone number () （Nurhazrina Binti Noordin) OR Associate Professor Dr Maria Justine ().

Research Risk

There are no known risks associated with this research towards all of face validators. Time consuming is the main risk for the face validators to complete the validation process.

Confidentiality

Your information will be kept confidential by the investigators and will not be made public unless disclosure is required by law.

By signing this consent form, you will authorize the review of records, analysis and use of the data arising from this research.

Consent Form[1]

To become a subject in the research, you or your legal guardian is required to sign this Consent Form.
I herewith confirm that I have met the requirement of age and am capable of acting on behalf of myself / as[2] a legal guardian as follows:

1. I understand the nature and scope of the research being undertaken.
2. I have read and understood all the terms and conditions of my participation in the research.
3. All my questions relating to this research and my participation therein have been answered to my satisfaction.
4. I voluntarily agree to take part in this research, to follow the study procedures and to provide all necessary information to the investigators as requested.
5. I may at any time choose to withdraw from this research without giving any reason.
6. I have received a copy of the Subjects Information Sheet and Consent Form.
7. Except for damages resulting from negligent or malicious conduct of the researcher(s), I hereby release and discharge UiTM and all participating researchers from all liability associated with, arising out of, or related to my participation. I agree to hold them harmless from any harm or loss that may be incurred by me due to my participation in the research.

_______________________________________ Name of Subject/Legally authorized representative (LAR) Signature

_______________________________________ I.C No Date

_______________________________________ Name of Witness[3] Signature

_______________________________________ I.C No Date

_______________________________________ Name of Consent Taker Signature

_______________________________________ I.C No Date

[1] Original signed copy is to be retained by the Principal Investigator.
[2] Delete whichever is not applicable.
[3] A witness is only required for oral consent.

Borang Maklumat Subjek
(Peserta)

Tajuk Penyelidikan

Kajian untuk menentukan kesan PTICOPE terhadap kapasiti intrinsik (kognitif, lokomotor, psikologi, kecergasan, pendengaran, dan penglihatan) dalam kalangan warga emas yang tinggal di dalam komuniti.

Pengenalan Penyelidikan

Penuaan penduduk adalah isu global yang memberi kesan besar kepada individu, masyarakat, dan sektor kerajaan. Salah satu kesan utama penuaan ialah penurunan kapasiti intrinsik, yang merangkumi enam domain utama: lokomotor, psikologi, kecergasan, kognitif, pendengaran, dan visual. Pada tahun 2017, Pertubuhan Kesihatan Sedunia (WHO) memperkenalkan rangka kerja yang menggabungkan kapasiti intrinsik dan keupayaan fungsi, dikenali sebagai Integrated Care for Older People (ICOPE). Namun, beberapa kajian terbaru yang menggunakan rangka kerja ICOPE mendapati ia tidak sesuai untuk digunakan di Malaysia.

Untuk mengatasi masalah ini, kajian ini sedang mengembangkan satu intervensi baru dalam bentuk buku kerja yang dipanggil Penjagaan Berpusatkan Individu Berintegrasi Untuk Orang Tua (PTICOPE). PTICOPE menggabungkan intervensi senaman, nasihat, dan pemantauan untuk warga emas yang dipimpin oleh ahli fisioterapi. Buku kerja ini merangkumi visual senaman dan carta pemantauan untuk membantu peserta memantau rutin harian mereka, termasuk senaman, pengambilan diet, dan pemantauan penilaian rumah. Selain itu, kajian ini juga bertujuan untuk menilai perubahan dalam tahap kapasiti intrinsik sebelum(pre-minggu pertama) dan selepas (post-minggu ke-enam) pelaksanaan PTICOPE pada peserta.

PTICOPE disasarkan kepada warga emas, melibatkan interaksi dan pemantauan berkala peserta bersama komuniti dan profesional penjagaan kesihatan. Ini dilakukan berdasarkan domain kapasiti intrinsik yang terjejas serta ketersediaan profesional penjagaan kesihatan yang berdekatan dengan kawasan kediaman peserta.

Tujuan Penyelidikan

Kajian ini bertujuan untuk menilai kesan PTICOPE terhadap kapasiti intrintik (fungsi kognitif, lokomotor [pergerakan], kecergasan, fungsi psikologi, fungsi penglihatan dan pendengaran) warga emas di komuniti.

Prosedur Penyelidikan

Jika anda bersetuju untuk mengambil bahagian dalam kajian ini, anda dikehendaki menandatangani borang persetujuan. Kemudian anda akan didaftarkan dalam salah satu kumpulan sama ada kumpulan

kawalan atau kumpulan intervensi. Kumpulan intervensi akan menerima buku kerja PTICOPE dengan panduan komprehensif tentang cara menggunakan buku kerja. Kumpulan kawalan akan diberi pengetahuan am tentang kepentingan kapasiti intrinsik yang baik dan nasihat tentang cara untuk meningkatkan setiap domain kapasiti intrinsik yang mungkin termasuk latihan dan nasihat untuk mendapatkan perkhidmatan penjagaan kesihatan yang berkaitan berdasarkan penemuan daripada pengukuran pertama (baseline). Latihan untuk kumpulan intervensi akan dijalankan kira-kira 3 sesi /3 kali seminggu selama 6 minggu. Tempoh setiap sesi untuk PTICOPE ialah 60 minit, dengan 5 minit aktiviti penyediaan (memanaskan badan dengan regangan), 10 minit diperuntukkan untuk setiap bahagian dalam intervensi yang mengandungi senaman iaitu dari 4 domain; kognitif, lokomotor, visual dan pendengaran. Selain itu, domain di bawah pemantauan iaitu 2 domain ; kecergasan dan pendengaran diperuntukkan selama 5 minit untuk setiap domain, dan 5 minit terakhir untuk berehat. Pengukuran awal (basseline) anda yang merangkumi butiran demografi, klinikal, antropometri dan butiran komposisi badan, kecergasan berfungsi dan keupayaan berfungsi akan dinilai sekali sahaja pada minggu pertama pengumpulan data awal. Tahap kapasiti intrinsik terdiri daripada 6 domain utama; psikologi, kecergasan, kognitif, pendengaran, visual dan lokomotor akan dinilai sebelum (minggu pertama) dan selepas (minggu keenam).

Penyertaan dalam Penyelidikan

Penyertaan anda dalam kajian ini adalah secara sukarela. Anda boleh menolak untuk mengambil bahagian dalam kajian atau anda boleh menarik diri daripada penyertaan dalam kajian pada bila-bila masa tanpa penalti.

Faedah Penyelidikan

Maklumat yang diperoleh daripada kajian ini akan memberi manfaat kepada penyelidik, diri sendiri, profesional penjagaan kesihatan dan individu lain dalam memahami faedah PTICOPE yang menggabungkan pasukan pelbagai disiplin dalam menguruskan isu penurunan kapasiti intrinsik dan menggalakkan komuniti penuaan kearah yang lebih sihat.
Sebaliknya, kami akan memaklumkan anda tentang penemuan daripada kajian ini terutamanya berkaitan tahap kapasiti intrinsik anda. Kami juga akan menasihati anda tentang penerusan latihan dan kemungkinan merujuk anda untuk mendapatkan nasihat pakar.
Jika anda mempunyai sebarang soalan mengenai kajian ini atau hak anda, sila hubungi penyiasat di nombor telefon ()Nurhazrina Binti Noordin) ATAU Profesor Madya Dr Maria Justine ()

Risiko Penyelidikan

Tiada risiko diketahui berkaitan dengan penyelidikan ini, namun, anda mungkin berasa sedikit letih selepas melakukan senaman dan ia akan berkurangan dengan rehat. Setiap sesi senaman memberi kesan risiko yang minimum kepada peserta dan tempoh rehat juga diperuntukkan sepanjang sesi.

Kerahsiaan

Maklumat anda akan dirahsiakan oleh penyelidik dan tidak akan didedahkan kepada umum melainkan pendedahan diperlukan oleh undang-undang.

Dengan menandatangani borang persetujuan ini, anda akan membenarkan semakan rekod, analisis dan penggunaan data yang timbul daripada penyelidikan ini.

Borang Persetujuan 1

Untuk menjadi subjek dalam penyelidikan, anda atau penjaga sah anda dikehendaki menandatangani Borang Persetujuan ini.

Saya dengan ini mengesahkan bahawa saya telah memenuhi syarat umur dan berkemampuan untuk bertindak bagi pihak saya sendiri / sebagai penjaga yang sah seperti berikut:

1. Saya memahami sifat dan skop penyelidikan yang dijalankan.
2. Saya telah membaca dan memahami semua terma dan syarat penyertaan saya dalam penyelidikan.
3. Semua soalan saya yang berkaitan dengan penyelidikan ini dan penyertaan saya di dalamnya telah dijawab dengan kepuasan saya.
4. Saya secara sukarela bersetuju untuk mengambil bahagian dalam penyelidikan ini, mengikuti prosedur kajian dan memberikan semua maklumat yang diperlukan kepada penyiasat seperti yang diminta.
5. Saya boleh pada bila-bila masa memilih untuk menarik diri daripada penyelidikan ini tanpa memberi sebarang sebab.
6. Saya telah menerima salinan Lembaran Maklumat Mata Pelajaran dan Borang Persetujuan.
7. Kecuali untuk kerosakan akibat daripada kecuaian atau kelakuan jahat penyelidik, saya dengan ini melepaskan dan melepaskan UiTM dan semua penyelidik yang mengambil bahagian daripada semua liabiliti yang berkaitan dengan, timbul daripada, atau berkaitan dengan penyertaan saya. Saya bersetuju untuk memastikan mereka tidak berbahaya daripada sebarang bahaya atau kerugian yang mungkin ditanggung oleh saya disebabkan penyertaan saya dalam penyelidikan.

Nama Subjek/Wakil yang diberi kuasa secara sah (LAR) Tandatangan

IC

Tarikh

Nama Saksi[3] Tandatangan

IC

Tarikh

Nama Pengambil Persetujuan Tandatangan

IC

Tarikh

1 Salinan asal yang ditandatangani hendaklah disimpan oleh Ketua Penyelidik.
2 Padamkan mana-mana yang tidak berkenaan.
3 Seorang saksi hanya diperlukan untuk persetujuan lisan.

Subject Information Sheet

Research Title

To determine the effects of PTICOPE on intrinsic capacity (cognitive, locomotors, psychology, vitality, hearing and vision) among older persons living in the community.

Introduction of Research

Aging populations are a global issue that significantly impacts individuals, communities, and government sectors. One of the main effects of aging is the decline in intrinsic capacity, which encompasses six major domains: locomotors, psychological, fitness, cognitive, auditory, and visual. In 2017, the World Health Organization (WHO) introduced a framework that integrates intrinsic capacity and functional ability, known as Integrated Care for Older People (ICOPE). However, several recent studies using the ICOPE framework have found it unsuitable for use in Malaysia.

To address this issue, this study is developing a new intervention in the form of a workbook called Integrated Person-Centered Care for Older People (PTICOPE). PTICOPE combines exercise interventions, advice, and monitoring for the elderly, led by a physiotherapist. This workbook includes exercise visuals and monitoring charts to help participants track their daily routines, including exercise, dietary intake, and home assessment monitoring. Additionally, this study aims to assess changes in the level of intrinsic capacity before (pre-first week) and after (post-sixth week) the implementation of PTICOPE on participants.

PTICOPE is targeted at the elderly, involving regular interaction and monitoring of participants with the community and healthcare professionals. This is done based on the affected domains of intrinsic capacity and the availability of healthcare professionals near the participants' residential areas.

Purpose of Research

This study aims to evaluate the effects of PTICOPE on the intrinsic capacity (cognitive function, locomotor [movement], vitality, and psychological function, visual and auditory functions) of older people in the community.

Research Procedure

If you agree to participate in the study, you will be required to sign the informed consent form. Then you will be enrolled in either control group or intervention group. The intervention group will receive the PTICOPE work-book with comprehensive guides on how to use the work-book. The control group will be given a general knowledge about the importance of good IC and advice on how to enhance each domain of IC that may include exercises and advice to seek relevant healthcare services based on findings from baseline IC capacity measurements. The training will be conducted approximately 3 sessions/3 times a week for 6 weeks. The duration of each session for PTICOPE is 60 minutes, with 5 minutes of preparation activity (warming up with stretches), 10 minutes allocate for each section in our interventions with exercise which contains of 4 domains; cognitive, locomotors, visual and hearing while monitoring domains; vitality and hearing allocated for 5 minutes, and the last 5 minutes of relaxation. Your baseline

which includes demographic, clinical details, anthropometry and body composition details, functional fitness, and functional ability will be assessed one time only during the first week of baseline data collections. Intrinsic capacity level consist of 6 main domains; psychological, vitality, cognitive, hearing, visual and locomotors will be assessed pre(first week) and post (sixth weeks).

Participation in Research

Your participation in this study is entirely voluntary. You may refuse to take part in the study or you may withdraw yourself from participation in the study at any time without penalty.

Benefit of Research

The information obtained from this study will benefit researchers, yourself, healthcare professionals, and other individuals in their future understanding the benefits of person-centered approach which combine of multidisciplinary team in managing the decline in intrinsic capacity and promoting a healthy ageing community. On the other hand, we will inform you about your findings from this study especially with regards to your intrinsic capacity level. We will also advise you on continuation of the exercise and possibility on referring you for the expert advice.

If you have any question about this study or your rights, please contact the investigators at telephone number () （Nurhazrina Binti Noordin) OR Associate Professor Dr Maria Justine ().

Research Risk

There are no known risks associated with this research, however, you may feel slightly tired after doing the exercise and it will reduce with rest. Each exercise session develop with minimal risk impact to participants and rest period were also allocate throughout the sessions.

Confidentiality

Your information will be kept confidential by the investigators and will not be made public unless disclosure is required by law. By signing this consent form, you will authorize the review of records, analysis and use of the data arising from this research.

Consent Form[1]

To become a subject in the research, you or your legal guardian is required to sign this Consent Form.
I herewith confirm that I have met the requirement of age and am capable of acting on behalf of myself / as[2] a legal guardian as follows:

1. I understand the nature and scope of the research being undertaken.
2. I have read and understood all the terms and conditions of my participation in the research.
3. All my questions relating to this research and my participation therein have been answered to my satisfaction.
4. I voluntarily agree to take part in this research, to follow the study procedures and to provide all necessary information to the investigators as requested.
5. I may at any time choose to withdraw from this research without giving any reason.
6. I have received a copy of the Subjects Information Sheet and Consent Form.
7. Except for damages resulting from negligent or malicious conduct of the researcher(s), I hereby release and discharge UiTM and all participating researchers from all liability associated with, arising out of, or related to my participation. I agree to hold them harmless from any harm or loss that may be incurred by me due to my participation in the research.

____Name of Subject/Legally authorized representative (LAR) Signature

____ I.C No Date

____Name of Witness[3] Signature

____ I.C No Date

____Name of Consent Taker Signature

____ I.C No Date

[1] *Original signed copy is to be retained by the Principal Investigator.*
[2] *Delete whichever is not applicable.*
[3] *A witness is only required for oral consent.*
